# The relationship between learning organization and organizational commitment among nursing managers in educational hospitals of Isfahan University of Medical Sciences in 2008-9

**Published:** 2010

**Authors:** Maryam Yaghoubi, Ahmad Reza Raeisi, Mina Afshar, Mohammad Hossein Yarmohammadian, Akbar Hasanzadeh, Marzi Javadi, Maryam Ansary

**Affiliations:** * Student, Management of Healthcare Services, Researcher of Health Management and Economic Research Center, Isfahan University of Medical Sciences, Isfahan, Iran; ** Health Information Management, Health Management and Economic Research Center, Isfahan University of Medical Sciences, Isfahan, Iran; *** Instructor of Medical Library and Information, Isfahan University of Medical Sciences, Isfahan, Iran; **** Educational Planning, Head of Health Management and Economic Research Center, Isfahan University of Medical Sciences, Isfahan, Iran; ***** Instructor, Department of Vital Statistics, School of Health, Isfahan University of Medical Sciences, Isfahan, Iran; ****** Student, Philosophy of Education, Isfahan University of Medical Sciences, Isfahan, Iran

**Keywords:** Learning organizations, personnel management, administration, nurses

## Abstract

**BACKGROUND:**

Old methods of administrating can’t cover the rapid changes of today. These changes redounded new organizations like learning organizations to be formed. The purpose of this research was to study the relationship between learning organization and organizational commitment among nursing managers.

**METHODS:**

This was a descriptive analytic survey. The population of study included 90 nursing managers of 9 educational hospitals. Data gathering was done via learning organizational (LO) and organizational commitment (OC) questionnaires. Data analysis was done using SPSS software.

**RESULTS:**

The mean score of LO was 56.9 ± 18.1 among nursing mangers, and the mean score of OC was 62.3 ± 10.1. In general, there was a significant relationship between LO and OC and there was a significant relationship between LO and job experience based on ANOVA test.

**CONCLUSIONS:**

In today’s changing environment of very rapid changes which have been seen in different areas of science and technology and the increasing complexity and dynamics of environmental factors, only organizations with active adaptation (dynamic equilibrium) can survive and remain capable of growth. This aim can be fulfilled just in learning organizations.

Science and technology development and expansion of the business environment caused working environments to be competitive and full of challenges.^1^ A good workplace is believed to produce higher quality products, support more innovation, have the ability to attract more talented people, and experience less resistance to change and lower turnover costs, all of which translate directly into a better bottom line.^2^

New paradigm has been appeared that survival for many organizations is very difficult, so those organizations are more successful that learn earlier, better and faster than competitors. This is the reason why the concept of learning organizations has been raised in recent years.^1^ In today’s changing environment that very rapid changes are seen in different areas of science and technology and the increasing complexity and dynamics of environmental factors, the only organizations that can survive and remain capable of growth are those with active adaptation (dynamic equilibrium). Learning organization is an organization where people constantly increase their capabilities. The new patterns of thinking develop and staffs learn how to learn together.^3^ Learner organization is an organization that created the power of obtaining and transferring of knowledge and changes behaviors based on new knowledge and insights.^4^ This organization focuses on model development and applying knowledge and information capabilities in order to create impact and value of information.^5^

The idea of learning organization was popularized by Peter Senge (1990); he stated that at the core of the learning organization are five essential learning disciplines: personal mastery, mental models, shared vision, team learning, and systematic thinking; that may be briefly described as follow.

***Personal mastery*** is the discipline of continually clarifying and deepening our personal vision, focusing on our energies, developing patience, and seeing reality objectively.

***Mental models*** are deeply ingrained assumptions, generalizations, or even pictures or images that influence how we understand the world and how we take action.

***The practice of shared vision*** involves the skills of unearthing shared “pictures of the future” that foster genuine commitment and enrollment rather than compliance.

***The discipline of team learning*** starts with “dialogue,” the capacity of members of a team to suspend assumptions and enter into a genuine “thinking together.”

***Systematic thinking*** is based on system dynamics; it is highly conceptual; it provides ways of understanding practical business issues.^6^

One of the effective factors in human resources in hospitals is organization commitment (OC). There are different definitions for OC like other concepts in organizational behaviors. Of course the most common is considering it as “a kind of emotional dependency in organization” or “a kind of loyalty feeling to organization”.^7^,^8^

Someone who is completely committed gets his/her identity from organization, participates in programs and enjoys being a member of the organization.^7^

Many researchers have proved the positive effects of organization commitment on performance of organization. Staffs who have less commitments toward their job have more absence and may quit more than others.^9^,^10^

On the other hand researches showed that OC is an effective factor on staffs’ job satisfaction.^11^

Also organization outcome will be increased if the commitment goes up.^12^,^13^

High commitment can determine the effectiveness of the staffs in an organization.^14^ Dealing with organizational commitment Meyer et al (1997) divided organization commitment to three kinds: affective, continuance, normative.^9^ According to Meyer et al, three component model of commitment, prior research indicated that there are three “mind sets” which can characterize an employee’s commitment to the organization.

***Affective Commitment (AC)*** is defined as the employee’s positive emotional attachment to the organization. An employee who is affectively committed strongly identifies with the goals of the organization and desires to remain a part of the organization. This employee commits to the organization because he/she “wants to.” In developing this concept, Meyer et al drew largely on Mowday, Porter, and Steers’s (1982) concept of commitment, which in turn drew on earlier work by Kanter (1968).

***In Continuance Commitment (CC)*** the individual commits to the organization because he or she perceives high costs of losing organizational membership (cf. Becker’s 1960 “side bet theory”), including economic costs (such as pension accruals) and social costs (friendship ties with co-workers) that would be incurred. The employee remains a member of the organization because he/she “has to.”

***In Normative Commitment (NC)*** the individual commits to and remains with an organization because of feelings of obligation. These feelings may derive from many sources. For example, the organization may have invested resources in training an employee who then feels a “moral” obligation to put forth effort on the job and stay with the organization to “repay the debt.” It may also reflect an internalized norm, developed before the person joins the organization through family or other socialization processes, that one should be loyal to one’s organization. The employee stays with the organization because he/she “ought to.”^9^,^15^

Study conducted by Wang (2005) in the University of Minnesota showed that the components of learning organization consist of: participation, team learning, creating a systematic thinking, culture of learning organization and collaboration. Leadership strategy has a correlation with organizational commitment. He studied organization commitment in its three dimensions: affective, normative and continuance. These components had a relationship with the dimensions of organizational commitment.^16^

Egan et al (2004) showed that creating a culture of learning organization and job satisfaction and organizational commitment are positively related.^17^

A study conducted by Jeong et al (2007) showed that there is a relationship between the establishment of principles of organization and effectiveness of nurse managers (629 people). This cross-sectional study has been done in 9 hospitals in South Korea to measure the effectiveness of nurse managers, job satisfaction and organizational commitments. The results indicated that the establishment of the principles of learning organization and organizational commitment are related. These principals have increased commitment up to 24.9% and improved job satisfaction up to 22.6% in nurse managers. Principles of factors of this research are learning organization of Peter Senge’s.^18^

Study done by Yarmohammadzadeh (2007) showed there is a positive relation between managers and assistants with components of learning organization. In addition, there was no meaningful relation between managers and assistants ideas considering their experience and level of education to serve them significant difference.^19^

Malekpour (2004) in his research entitled: “survey on organizational culture and organizational commitment” done on staffs of hospitals in Isfahan University of medical sciences concluded that discipline, work experience, level of education and type of employment affected organizational commitment in staffs.^20^

Pak et al (2008) indicated that organizational learning is equally important in explaining organizational commitment, job satisfaction and work performance. At the same time, organizational learning, job satisfaction and organizational commitment are also equally important in explaining work performance among the public service managers.^21^

Joo (2009) concluded that organizational learning culture, proactive personality, and perceived job complexity accounted for 44% and 54% of the variances in organizational commitment and intrinsic motivation, respectively. In addition, proactive personality moderated the relationship between organizational learning culture and organizational commitment.^22^

Yaghoubi (2007) in a research entitled “the study on relationship between organizational commitment and job stress of Medical Sciences hospitals managers” showed that the mean score of organizational commitment (67 ± 13.7) is equal among men and women. So the relationship between the amount of commitment and sex is not significant. Organizational commitment among managers with a high work experience was more.^5^

Škerlavaj et al (2007) showed that OLC has a positive direct impact on all three aspects of non-financial performance included in the model: performance from the employee, customer, and supplier perspectives. The effect of organizational learning culture on financial performance is still positive, but indirect (through non-financial performance from the employee perspective).^23^

Steers (1977) found that there hadn’t been any significant correlation between OC and staffs’ level of education similar to this study. And there was a significant correlation between age and OC.^24^

Although organizational learning theory and practice have been clarified by practitioners and scholars over the past several years, there is much to be explored regarding interactions between organizational learning culture and employee learning. Especially in health care systems, for example hospitals. As Nurses, as the largest human resource element of health care systems, have a major role in providing ongoing, high quality care for patients^25^ and since nurses are 24-hour health service providers on the front line of contact with patients, and are essential to hospital operations, nurses’ occupational health is a major hospital management issue.^26^ So, the objective of the study was to determine the relationship between the learning organization and organizational commitment among nursing managers.

## Methods

This was a descriptive correlation (or relationship) survey. Study population was all 90 nursing managers employed in teaching hospitals of Isfahan University of Medical Sciences from October 2008 to February 2009. The managers were nursing managers (head nurses and supervisors). Meyer and Allen’s questionnaire was used to collect information for organizational commitment.^8^ For the learning organization, questionnaire was made based on principles of Peter Senge (researcher made).

Content validity was considered for instrument validity. Reliability was calculated via Cronbach’s Alpha (r = 0.85). Data analysis was done by SPSS software version 13 using ANOVA, t test and chi-square test.

## Results

Demographic results of the study show that 0.4% of sample population was male and was 96% female. From the education point of view, 21% had master degree and 79% had BS degree. Also 10% of sample population was between 20 and 30 years old, 34.2% between 31 and 40, 51% between 41 and 50 and 4.8% between 51 and 60; actually half of them were between 40-50 years old. From the experience point of view, 4.6% had less than 5 years of job experience, 15% had 5-10 years of job experience, 32.4% had 11-15 years, 25.2% had 16-20 years and 22.8% had 21-30 years of job experience. The following results have been revealed according to findings considering the main object of the project: generally, the mean score of organizational commitment was 62.3 (10.1). Affective commitment had the highest amount 67.3 (16.3) and continuous commitment had the least 59.5 (9.9). (Table 1)

**Table 1:** Means of organizational commitment and its dimension in managers of Isfahan educational hospitals

Variable	Organizational commitment	Affective commitment	Continuous commitment	Normative commitment
Mean	62.3	67.3	59.5	60.3
Standard deviation	10.1	16.3	9.9	13.5

The mean score of learning organization was 56.9 (18.1).

Personal mastery had the highest amount 67.3 (16.3) and systematic thinking had the least 59.5 (9.9) (Table 2).

**Table 2:** Means of learning organization and its dimension in managers of Isfahan educational hospitals

Variable	Learning organization	Personal mastery	Mental model	Building shared vision	Systematic thinking	Team learning
Mean	56.9	63.2	55	58	52.6	55.7
Standard deviation	18.1	18.4	24	18	21.5	20.8

There was no relationship between sex, age and education and organization commitment according to ANOVA statistical analysis test.

But, there was a meaningful relationship between organization commitment and work experience. The more was the work experience the more was the organization commitment. There was no relationship between sex, age, work experience and learning organization according to ANOVA statistical test results. There was meaningful relationship between learning organization and education. In general there was a significant relationship between learning organization and organization commitment (Table 3).

**Table 3:** Relationship between learning organization and organizational commitments in managers of Isfahan educational hospitals

Variable	Normative	Continuance	Affective	Organization commitment
Learning organization	r = 0.009	r = 0.299	r = 0.085	r = 0.85
	p = 0.913	p = 0.001	p = 0.003	p = 0.026

## Discussion

As hospital deals with patient’s life, the managers there should be completely expert, also should have high commitment due to the importance of atmosphere.

Learning organization is not planned just for developed societies; all organizations need to go through it in order to survival and persistence. Organizational change and development is essential for Iranian service and industry organizations strongly. Especially hospitals because of vitality, variety, close relationship with customers (patients) and type of services that provide.

Nursing, as one of the oldest professions, is undergoing changes. In an attempt to provide better health care to the community, health care institutions are also undergoing changes. These transformations have had significant impact on nurses, administrators, health care organizations and society at large. The provision of better management of nurses, in the light of changes in the health industry, were discussed from the cultural, interpersonal and systems thinking perspectives of organizational learning.

Regarding the role of nurse managers and administrators, especially in hospitals, there is a strong need to increase their commitment in order to move towards the learning organization.

The managers who have more commitment have more loyalty in their job, and remain in organization and work more for it. Managers should keep their own and their personnel commitment and loyalty and improve them.^14^

The result of study done by Mosaddeghrad about “OC among IUMS staffs and it’s relationship with job satisfaction” showed that the affective commitment had been in a good level and normative and continuance commitments to be in a moderate level. He also stated that there had been a significant correlation between OC and some other factors such as the level of education, age, salary and job experience; so that the staffs who have higher education or are older than 30 or get more money, have more commitment in comparison with others.^7^ There are similar results in the current paper. Malekpour also considered the relationship between organizational culture and OC and concluded that age, field, job level, work experience, education and the kind of employment influence on OC in staffs of educational hospitals of Isfahan.^20^ This paper had the similar results about job experience.

Also, Yaghoubi (2006) in her research has found that today’s organizations need new and effective managerial skills to be able to achieve their goals. Results of our research revealed that the organizational commitment was in an acceptable level in the hospitals of Isfahan University of Medical Sciences. The affective commitment was higher than the continuance and normative commitment; because manager which have higher level of affective commitment stay in the organization as they “like” to stay. Finally, it is obvious that commitment is an effective factor on personnel’s performance so it must be noticed more than before and should be improved in our organizations. And moving toward a learning organization is one of the ways for increasing organizational commitment.

In the study conducted by Wang (2005), there was a correlation with organizational commitment. He studied organization commitment in its three dimensions: affective, normative and continuance. These components had a relationship with the dimensions of organizational learning. There are similar results in the current paper.

Jeong (2007) showed that there is a relationship between the establishment of principles of learning organization and organizational commitment of nurse managers. Principles of factors of this research are learning organization of Peter Senge’s.^18^ There are similar results in the current paper.

Finally, learning organization initiated by Senge (1990) in the last decade of the twentieth century and has provided a new vision for managing organizations effectively. Numerous business organizations and a few health care organizations have used it as an ideal approach for dealing with the rapid and unpredictable changes occurring in our modern world. Hospitals like other organizations in dealing with the rapid changes have found that learning organization principles are useful and necessary.

Nurses’ manager can use the principles of learning organization to enhance organizational effectiveness. Intervention programmers that integrate and strengthen shared vision and team learning may be useful to enhance organizational effectiveness. Further research is required to identify other factors related to the principles of learning organization.

The Authors declare that have no conflict of interest in this study and ethical committee approved the study.
